# A New Species in *Pseudophialophora* From Wild Rice and Beneficial Potential

**DOI:** 10.3389/fmicb.2022.845104

**Published:** 2022-03-11

**Authors:** Jia-Nan Zhu, Yi-Jun Yu, Meng-Di Dai, Yu-Lan Zeng, Xuan-Jun Lu, Ling Wang, Xiao-Hong Liu, Zhen-Zhu Su, Fu-Cheng Lin

**Affiliations:** ^1^State Key Laboratory for Managing Biotic and Chemical Treats to the Quality and Safety of Agro-Products, Institute of Biotechnology, Zhejiang University, Hangzhou, China; ^2^Department of Agriculture and Rural of Zhejiang Province, Hangzhou, China; ^3^State Key Laboratory for Managing Biotic and Chemical Treats to the Quality and Safety of Agro-Products, Institute of Plant Protection and Microbiology, Zhejiang Academy of Agricultural Sciences, Hangzhou, China

**Keywords:** endophytic fungi, *Pseudophialophora*, symbiosis, growth promotion, disease resistance

## Abstract

Wild rice (*Oryza granulata*) is a natural resource pool containing abundant unknown endophytic fungi species. There are few reports on the endophytic fungi in wild rice. Here, one isolate recovered from wild rice roots was identified as a new species *Pseudophialophora oryzae* sp. nov based on the molecular phylogeny and morphological characteristics. Fluorescent protein-expressing *P. oryzae* was used to monitor the fungal colonization pattern. Hyphae invaded the epidermis to the inner cortex but not into the root stele. The inoculation of *P. oryzae* promoted the rice growth, with the growth parameters of chlorophyll content, shoot height, root length, fresh shoot weight, fresh root weight and dry weight increasing by 24.10, 35.32, 19.35, 90.00, 33.3, and 79.17%, respectively. *P. oryzae* induced up-regulation of nitrate transporter *OsPTR9* and potassium transporter *OsHAK16* by 7.28 ± 0.84 and 2.57 ± 0.80 folds, promoting nitrogen and potassium elements absorption. In addition, *P. oryzae* also conferred a systemic resistance against rice blast, showing a 72.65 and 75.63% control rate in sterile plates and potting conditions. This systemic resistance was mediated by the strongly up-regulated expression of resistance-related genes *NAC*, *OsSAUR2*, *OsWRKY71*, *EL5*, and *PR1*α. Since *P. oryzae* can promote rice growth, biomass and induce systemic disease resistance, it can be further developed as a new biogenic agent for agricultural production, providing a new approach for biocontrol of rice blast.

## Introduction

Endophytic fungi have been found colonizing all plant species and grow symptomatically in host plant tissues (Carroll, 1988). Endophytic fungi confer benefits to the host plants by promoting growth, enhancing resistance to biotic and abiotic stresses (Sieber, 2002), and improving the host’s ecological adaptability (Schulz and Boyle, 2005; Bertolazi et al., 2019; Domka et al., 2019; Vergara et al., 2019; White et al., 2019). Endophytic fungi promote plant growth and development by increasing nutrient intake of nutrient elements by the host plants (Rana et al., 2020). Phytohormones play as messengers to control plant growth and development (Aly et al., 2010). Certain endophytes synthesize phytohormones, such as indole-3-acetic acid (IAA), gibberellins (GAs), and cytokinins, to promote host plant growth (You et al., 2013; Khan A. L. et al., 2014; Khan A. R. et al., 2014). In addition, endophytic fungi also play essential roles on improving plant disease resistance. *Harpophora oryzae* isolated from the wild rice roots possessed biocontrol potential to rice blast (Yuan et al., 2010; Su et al., 2013).

Magnaporthales is an order of Sordariomycetes, Ascomycota (Zhang et al., 2011; Luo et al., 2015). About 50% of these species are pathogens of monocotyledons, such as rice, maize and wheat (Kirk et al., 2008; Luo and Zhang, 2013). *Pseudophialophora* is a newly established genus in Magnaporthaceae of Magnaporthales (Luo et al., 2014). This study isolated and identified a new species, endophytic fungus *P. oryzae* sp. nov, from the wild rice roots based on phylogenetic and molecular analysis. We monitored the colonization pattern of *P. oryzae* in rice roots by transferring the GFP fluorescence label, then detected the biomass of *P. oryzae* in vivo by real-time PCR. The effect of *P. oryzae* on promoting plant nutrient absorption and disease resistance was further investigated. This work provides a scientific basis for *P. oryzae* as biological hormones, biological control agents and biological fertilizers.

## Materials and Methods

### Fungal Isolation and Cultivation

Wild rice (*Oryza granulata*) samples were collected from Xishuangbanna, Yunnan province, southwest of China, in November 2019. The isolation method of endophytic fungi referred to Yuan’s method (Yuan et al., 2010). Briefly, the healthy rice roots were gently rinsed with tap water, then immersed in 75% ethanol for 30 s and 1% sodium hypochlorite for 10 min. Subsequently, the roots were rinsed with sterile distilled water three times and cut into approximately 5 mm long segments. The segments were then transferred into a malt extract agar (MEA) medium (2% malt extract, 2% agar). The plates were incubated at 25°C in darkness. Fungal cultures were isolated and purified, saved on potato dextrose agar (PDA) slope (Yuan et al., 2010).

### DNA Extraction, PCR Amplification, and Phylogenetic Analyses

Fungal DNA was extracted by DNA extraction method (Chi et al., 2009). Six genes, internal transcribed spacer (ITS), large subunit (LSU) and small subunit (SSU) of ribosomal RNA genes, DNA replication licensing factor (MCM7), the largest subunit of RNA polymerase II (RPB1), and translation elongation factor 1-α (TEF1-α) genes, were amplified for identification (Zhang et al., 2011; Luo and Zhang, 2013). Primers are listed in Supplementary Table 1. PCR amplification refers to the method of Zhang et al. (2011). PCR products were sequenced by ABI3730 (Tsingke company, Beijing), and the sequencing results were compared with the BLAST sequence on the national center for biotechnology information (NCBI) website. All reference strain names used for phylogenetic analysis and isolate numbers, sources, hosts, and GenBank accession numbers were listed in Table 1 (Luo and Zhang, 2013; Luo et al., 2014). The partial sequences of strain P-B313 were submitted to the GenBank and obtained GenBank accession numbers (Table 1). Sequences of each gene were aligned with Clustal X 2.1 (Thompson et al., 1997) and manually corrected with Genedoc (Yuan et al., 2010). A six-gene dataset was generated by connecting the individual sequence alignments. JModel Test 2.1.7 (Posada, 2008) was used to calculate the best-fit nucleotide substitution models by computing likelihood scores and calculating AIC. *Cryphonectria parasitica* was chosen as the outgroup taxon. Bayesian inference (BI) trees were constructed in MrBayes v3.2.6 (Ronquist et al., 2012), using the optimal nucleotide substitution model. A total of 100,000 trees were produced. The latter 37,500 trees were selected to calculate the posterior probability values of each branch in the consensus tree. Maximum-likelihood (ML) analysis with the selected optimal model was executed in IQ-Tree (Nguyen et al., 2015). Branch support was evaluated by 1000 bootstraps replicates.

**TABLE 1:** Species name, isolate ID, source, host, and GenBank accession numbers of the fungi used in this study.

Species name	Isolate ID	Source	Host	SSU	ITS	LSU	MCM7	RPB1	TEF1
***Pseudophialophora oryzae***	**P-B313**	**Yunnan, China**	***Oryza granulate***	**OL615103**	**OL614338**	**OL615091**	**OL657329**	**OL675673**	**OL675674**
*Magnaporthiopsis poae*	M47	NJ, United States	*Poa pratensis*	JF414860	JF414836	JF414885	JF710390	JF710433	JF710415
*Magnaporthiopsis rhizophila*	M23	Unknown	*Poa pratensis*	JF414858	JF414834	JF414883	JF710384	JF710432	JF710408
*Magnaporthiopsis incrustans*	M51	KS, United States	*Zoysia matrella*	JF414870	JF414846	JF414895	JF710389	JF710440	JF710417
*M. incrustans*	M35	Unknown	Unknown	JF414867	JF414843	JF414892	JF710386	JF710437	JF710412
*Magnaporthiopsis maydis*	M84	Unknown	Unknown	KM009208	KM009160	KM009148	KM009172	KM009184	KM009196
*M. maydis*	M85	Unknown	Unknown	KM009209	KM009161	KM009149	KM009173	KM009185	KM009197
*Magnaporthiopsis agrostidis*	BRIP 59300	United States	*Ultradwarf bermudagrass*	MF178145	KT364753	KT364754	MF178161	KT364755	KT364756
*Magnaporthiopsis cynodontis*	D29387-3	United States	*Ultradwarf bermudagrass*	MK458746	MK458730	MK458740	MK458750	MK458761	MK458756
*Magnaporthiopsis meyeri-festucae*	FF2	United States	*Ultradwarf bermudagrass*	MF178140	MF178146	MF178151	MF178156	MF178162	MF178167
*Magnaporthiopsis panicorum*	CM2s8	NJ, United States	*Panicum* sp.	KF689593	KF689643	KF689633	KF689603	KF689613	KF689623
*Gaeumannomyces graminis* var. *graminis*	M54	FL, United States	Unknown	JF414873	JF414848	JF414898	JF710394	JF710444	JF710419
*G. graminis* var. *graminis*	M33	FL, United States	*Stenotaphrum secundatum*	JF414871	JF710374	JF414896	JF710392	JF710442	JF710411
*G. graminis* var. *tritici*	M55	MT, United States	*Triticum* sp.	JF414875	JF414850	JF414900	JF710395	JF710445	JF710420
*G. graminis* var. *avenae*	CBS187.65	Netherlands	*Avena sativa*	JX134655	JX134668	JX134680	JX134708	JX134722	JX134694
*Buergenerula spartinae*	ATCC 22848	Unknown	*Spartina*	DQ341471	JX134666	DQ341492	JX134706	JX134720	JX134692
*Pseudophialophora schizachyrii*	AL3s4	NJ, United States	*Poaceae* sp.	KF689600	KF689650	KF689640	KF689610	KF689620	KF689630
*P. schizachyrii*	AL2m1	NJ, United States	*Schizachyrium* sp.	KF689599	KF689649	KF689639	KF689609	KF689619	KF689629
*P. panicorum*	CM3m7	NJ, United States	*Poaceae* sp.	KF689602	KF689652	KF689642	KF689612	KF689622	KF689632
*P. panicorum*	CM9s6	NJ, United States	*Panicum* sp.	KF689601	KF689651	KF689641	KF689611	KF689621	KF689631
*Pseudophialophora tarda*	WSF:14SW13	NJ, United States	*Dichanthelium acuminatum*	KP769823	KP769839	KP769831	KP784814	KP784822	KP784830
*P. tarda*	WSF:14RG48-2	NJ, United States	*Dichanthelium acuminatum*	KP769824	KP769840	KP769832	KP784815	KP784823	KP78483
*Pseudophialophora angusta*	WSF:14RG40	NJ, United States	*Dichanthelium acuminatum*	KP769825	KP769841	KP769833	KP784816	KP784824	KP784832
*Pseudophialophora dichanthii*	WSF:14RG82	NJ, United States	*Dichanthelium acuminatum*	KP769822	KP769838	KP769830	KP784813	KP784821	KP784829
*P. dichanthii*	WSF14RG72	NJ, United States	*Dichanthelium acuminatum*	KP769821	KP769837	KP769829	KP784812	KP784820	KP784828
*Pseudophialophora magnispora*	CM14RG38	NJ, United States	*Dichanthelium acuminatum*	KP769819	KP769835	KP769827	KP784810	KP784818	KP784826
*P. magnispora*	CM14RG50	NJ, United States	*Dichanthelium acuminatum*	KP769820	KP769836	KP769828	KP784811	KP784819	KP784827
*Pseudophialophora whartonensis*	WSF14RG66	NJ, United States	*Dichanthelium acuminatum*	KP769818	KP769834	KP769826	KP784809	KP784817	KP784825
*Pseudophialophora eragrostis*	CM20m5-2	NJ, United States	*Poaceae* sp.	KF689597	KF689647	KF689637	KF689607	KF689617	KF689627
*P. eragrostis*	CM12m9	NJ, United States	*Eragrostis* sp.	KF689598	KF689648	KF689638	KF689608	KF689618	KF689628
*Pyricularia grisea*	M82	Tichnor, AR, United States	*Digitaria* sp.	JX134656	JX134670	JX134682	JX134710	JX134724	JX134696
*Ophioceras commune*	M91	Yunnan, China	Rotten wood	JX134661	JX134675	JX134687	JX134715	JX134729	JX134701
*Nakataea oryzae*	M21	Japan	*Oryza sativa*	JF414862	JF414838	JF414887	JF710382	JF710441	JF710406
*Omnidemptus affinis*	ATCC 200212	QLD, Australia	*Panicum effusum* var. *effusum*	JX134660	JX134674	JX134686	JX134714	JX134728	JX134700
*Slopeiomyces cylindrosporus*	CBS 610.75	Unknown	Unknown	DQ341473	JX134667	DQ341494	JX134707	JX134721	JX134693
*Cryphonectria parasitica*	EP155	CT, United States	*Castanea dentata*	Genome data, Joint Genome Institute					

*Bold values represent GeneBank accession numbers of six genes of Pseudophialophora oryzae.*

### Morphological Observation and Genetic Transformation

Strain P-B313 was cultured in 150 mL potato dextrose broth (PDB) at 25°C 150 rpm for 3 days. The mycelia and conidia were then collected and observed under a microscope (Carl Zeiss Inc., Germany).

P-B313 fungal plug (5 mm × 5 mm) was fixed into 2.5% glutaraldehyde solution at 4°C overnight. Then the samples were rinsed with 0.1 M phosphate buffer (pH = 7) three times (15 min each time), fixed in 1% OsO_4_ for 2 h at 25°C, washed with phosphate buffer three times and dehydrated in a graded ethanol series. The samples were dried on HCP-2 critical point dryer (Hitachi, Japan) and coated. Finally, the samples were observed under SU-8010 scanning electron microscope (SEM) (Hitachi, Japan) (Liu X. H. et al., 2007).

The strain P-B313 was cultured in PDB for 3 days. And the conidia suspension with a concentration of 1 × 10^6^ spores/mL was collected. *Agrobacterium tumefaciens* strains containing PKD5-GFP vector with sulfonylureas resistance gene were mixed with P-B313 conidia suspension in equal volume (Lu et al., 2014). The transformants were screened on a defined complex medium (DCM) containing sulfonylurea (Dai et al., 2021). The fluorescence was detected by LSM880 confocal laser scanning microscope (Carl Zeiss Inc., Germany).

### Co-cultivation of Endophyte and Rice

Rice seeds of blast-susceptible rice cultivar CO-39 (*Oryza sativa*) were surface-sterilized in 70% ethanol for 5 min, in 1.0% sodium hypochlorite solution for 20 min rinsed repeatedly using sterile water. Rice seeds were then planted in half-strength Murashige and Skoog medium (Murashige and Skoog, 1962) for 3 days, then transferred into tissue culture bottles (8 cm in width, 50 cm in height) containing half-strength Murashige and Skoog in which 10 seedlings were inoculated. We then inoculated three fresh mycelium plugs (diameter 8 mm, 7-day-old) in each tissue culture vessel. Blank agar blocks were used as control.

### Quantification of Fungal Biomass in Rice Roots by Real-Time PCR

After 14 days of co-culture with GFP-tagged strain P-B313, the roots of the symbionts were collected and observed under an LSM880 confocal laser scanning microscope (Carl Zeiss Inc., Germany).

The fungus/plant DNA ratio (FPDR) was used to detect fungal infection in rice roots. The degree of fungal infection was determined by 2^–Δ^ *^Ct^* (Kenneth and Thomas, 2002), where ΔCt was the difference threshold value between strain P-B313 *Tef-1*α gene and rice *Actin* gene (Deshmukh et al., 2006; Deshmukh and Kogel, 2007). The specific primers were designed to be consistent with the *tef-1*α gene amplification primers. A total of 100 mg of root samples were collected at 5, 10, 15, and 20 days after inoculation (d.a.i.), respectively, according to Maciá-Vicente et al. (2009). The DNA was extracted using the nuclear plant genomic DNA kit (Tiangen, Beijing). The real-time PCR was performed in a total volume of 25 μL, including 10 ng of DNA, 12.5 μL of 2x SYBR Premix Ex Taq™ (Takara Bio Inc., Shiga, Japan), 1.25 μL of specific primer TEF1-F/R (or Actin-F/R for the rice *Actin* gene; Supplementary Table 1) and 10.25 μL of ddH_2_O. Melting curve analysis was performed. Ct values were measured by using the Realplex software 2.2.10.84.

### Endophytic Fertilizer Preparation and Pathogen Inoculation

Strain P-B313 was cultured in 150 mL PDB at 25°C 150 rpm for 3 days. The mycelium suspension was then inoculated into sterilized barley grains (150 mL/200 g) and fermented at 25°C for 15 days. The germinated rice seeds were planted into pots containing fermented fungal fertilizer (75 g fertilizer, 30 seeds per pot). The controls were rice seeds inoculated with sterile barley grains. After 14 days of co-culture, the growth parameters, such as the chlorophyll content, shoot length, root length, shoot fresh weight, fresh root weight, and dry weight, were determined. A total of 30 rice plants were measured in the control and treatment groups, respectively. The length of the longest root was measured.

The pathogen *Magnaporthe oryzae* Guy11 was cultured in a complete medium (CM) 10 days. Then the spores were collected and prepared into suspension with a concentration of 5 × 10^4^ spores/mL. The rice leaves were sprayed with spore suspension and incubated in the dark at 22°C for 2 days, at 25°C for 4 days (light 16 h/darkness 8 h). The lesion area rate and disease index were calculated. The disease index was investigated according to the Standard Evaluation System for Rice (SES) of the International Rice Research Institute (IRRI 2002) (Supplementary Table 2). The disease equation is as follows: disease index = Σ(diseased level leaf number × representative value) / (total leaf number × heavy disease representative value) × 100% (Li et al., 2020).

### Determination of Nutrient Elements

The rice leaves and roots were collected separately and dried to constant weight under −80°C, then ground into dry powder. A total of 0.5 g of dry powder sample was placed in the digestion tank with 5 mL concentrated nitric acid and 1 mL hydrogen peroxide, shake well and let it stand for 1 min before digestion. After digestion, the acid was heated on an electric stove. And after cooling, use 2% nitric acid to make the volume 200 mL. Finally, phosphorus (P), potassium (K), magnesium (Mg), and iron (Fe) were determined by ICP-OES (IRIS Intrepid II XSP, Thermo, United States). The nitrogen (N) content is determined by Kjeldahl method (Stafilov et al., 2020).

### Determination of Relative Expression Levels of Related Genes

After co-culture of strain P-B313 with rice for 14 days, rice plants were collected. Total rice RNA was extracted using TRIzol (Invitrogen, United States), followed by PrimeScript™ RT reagent Kit with gDNA Eraser (Perfect Real Time) (TaKaRa, Japan) kit for reverse transcription. The rice nutrition absorption-related genes *OsPTR9, OsAMT3;2, OsMRS2-8, OsPT4, OsHAK16, OsIRO2*, and *OsYSL15* and rice disease resistance-related genes *NAC, AOS, OsSAUR2, OsWRKY71, POX1, POX2, EL5, ERF4, PR1*α, and *PR1b* were measured by quantitative analysis. The real-time PCR was performed in a total volume of 20 μL, including template cDNA (five times diluted) 1 μL, 10 μL of 2x SYBR Premix Ex Taq™ (Takara, Japan), 1 μL of specific primer (Supplementary Table 1) and 7 μL of ddH_2_O. Reaction conditions: 95°C for 5 min, 40 cycles (95°C for 10 s, 60°C for 15 s), and the dissolution curve was set. The relative expression quantity of gene expression was calculated by 2^–ΔΔ*Ct*^ (Schmittgen and Livak, 2008).

### Statistical Analysis

Data were statistically analyzed by SPSS 16.0 version software (SPSS Inc., United States), expressed as mean ± standard deviation (SD). Graphs were created using GraphPad Prism 8.

## Results

### Morphological and Phylogeny Characteristics

**FIGURE 1:**
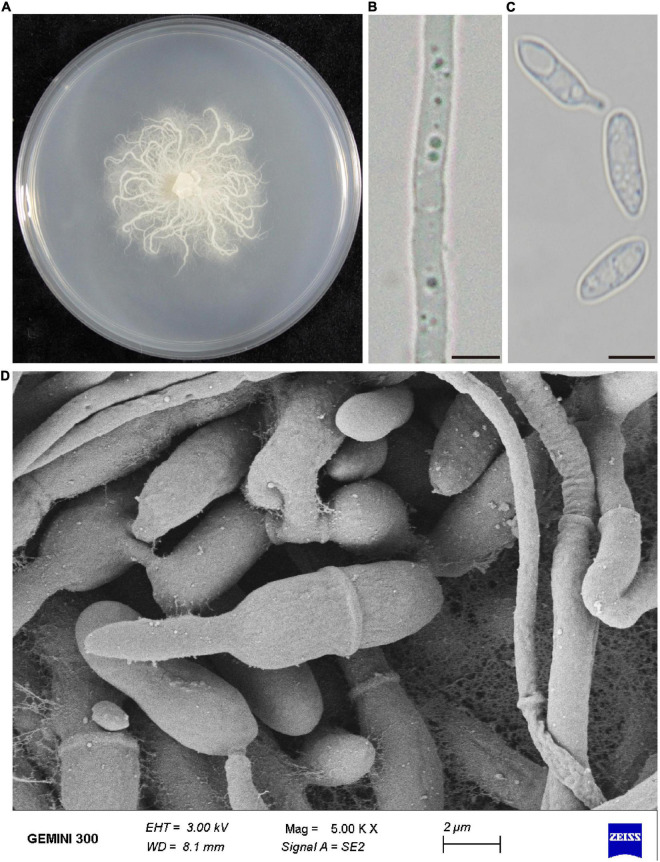
Morphological characteristics of *P. oryzae*. **(A)** Fungal colony on PDA after 7 days at 25°C. **(B)** Mycelia morphology under the optical microscope. Bar, 5 μm. **(C)** Conidia morphology under an optical microscope. Bar, 5 μm. **(D)** Scanning electronic micrographs of mycelia and conidia. Bar, 2 μm.

The morphology of the colony, hyphae and conidia were observed. Strain P-B313 grew slowly on PDA medium, and the colony diameter reached 4 cm after growing at 25°C for 7 days. Aerial mycelia were white, prostrating on the medium surface. Mycelia were 0.5–4.0 μm in width, with a septum. Conidiophores were solitary, no branching. Conidia were elliptic or dumbbell-shaped, 11–15 × 3.5–6.5 μm (Figure 1).

**FIGURE 2:**
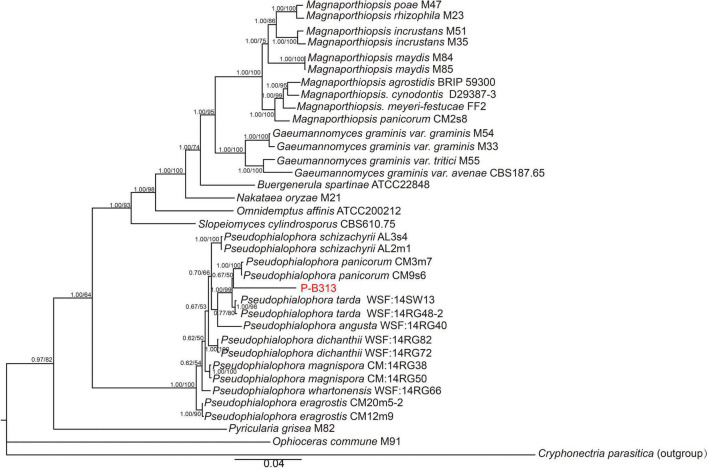
Phylogenetic tree of six genes combined. The tree was inferred from the combined ITS, SSU, LSU, TEF1, MCM7, and RPB1 sequence datasets. The topology of the tree is the result of BI method. The value on the branch is the BI posterior probability (BIPP)/ML bootstrap proportion (MLBP). The Bar indicates 0.04 base substitution sites.

We first blasted the similarity of the ITS sequence of the strain P-B313 on the NCBI website. The results showed that the identity between strain P-B313 and *Pseudophialophora* sp. (MK808146) was 99.4%. We conducted a phylogenetic analysis of strain P-B313 with the other related genus in Magnaporthaceae. It was found that there were 580 nucleotides in the ITS alignment, 869 in LSU, 1,032 in SSU, 926 in TEF1, 559 in MCM7, and 769 in RPB1. The 6-gene dataset involved 4,735 characters, including 925 parsimony informative, 722 variable and parsimony uninformative, and 3,088 constant. Calculated by jModel Test2.1.7, TN + F + R4 and TrN + I + G were selected as the optimal BI and ML analysis models. The two trees’ topological structures are similar using phylogenetic trees constructed by BI and ML methods. Only the BI tree is shown in Figure 2. Strain P-B313 belongs to the *Pseudophialophora* genus from the phylogenetic tree, but it exists in a separate clade independent of *Pseudophialophora panicorum* (Luo et al., 2014). In addition, the strain morphology and mycelium morphology of strain P-B313 and *P. panicorum* were quite different (Luo et al., 2014). Based on the molecular phylogeny and morphological, biological, and ecological characteristics, strain P-B313 was defined as a new species *P. oryzae* sp. nov (Collection Number: CCTCC M 2021504).

### Genetic Transformation, Colonization Pattern of *Pseudophialophora oryzae* in Rice Roots

**FIGURE 3:**
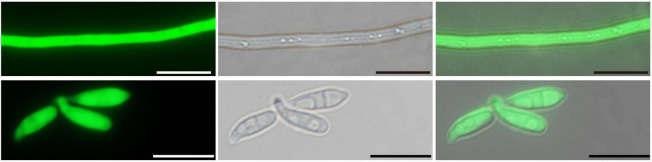
Laser scanning confocal microscopy of the GFP-expressed *P. oryzae* transformant. Hyphae and conidia showed constitutive GFP expression. Bar, 10 μm.

After five generations, intense green fluorescence was found to be uniformly distributed in the hyphae and conidiophores (Figure 3). The GFP-expressed transformant was selected as a candidate for further root inoculation.

**FIGURE 4:**
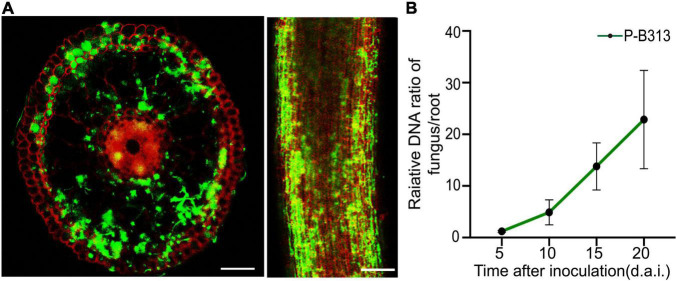
Colonization pattern of *P. oryzae* in rice roots. **(A)** GFP-tagged hyphae gradually extended from the epidermis to the endodermis in a root cross-section and longitudinal section. Bar, 5 μm. **(B)** Relative amounts of fungal DNA in rice roots at different time points (5, 10, 15, and 20 d.a.i.). A fungal colonization curve plotted with Mean ± SD is shown.

The colonization pattern was monitored using GFP-labeled *P. oryzae*. Transversely, the fungus entered the root epidermis and then invaded the inner cortical layer, finally colonized in the inner cortical layer. No hyphae approached the central part of the roots. Concomitantly, abundant hyphae preferred to colonize in the epidermis and outer cortex (Figure 4A).

The FPDR was measured simultaneously to assess fungal growth and the respective plant response. It was shown that an early moderate increase in the FPDR from 1.20 ± 0.18 to 4.90 ± 2.43 occurred within 10 d.a.i., followed by a significant increase to 22.85 ± 9.51 at 20 d.a.i. (Figure 4B).

### *Pseudophialophora oryzae* Promotes Rice Growth

**FIGURE 5:**
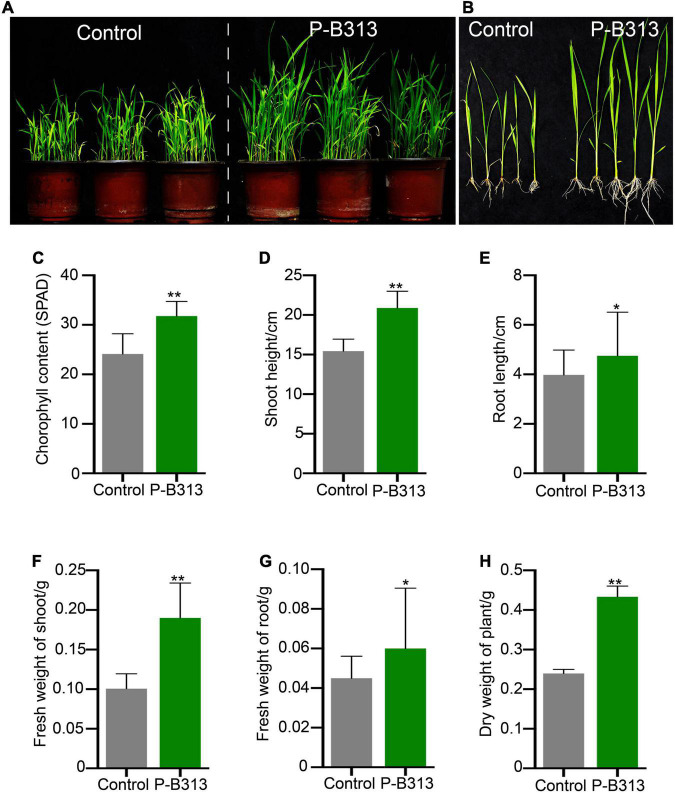
Effect of *P. oryzae* on the rice growth in pots. **(A,B)** The comparison of *P. oryzae*-treated plants with control in pots. **(C–H)** The comparison of *P. oryzae*-treated plants with non-treated control on the growth parameters includes the chlorophyll content, shoot height, root length, fresh shoot weight, fresh root weight, and plant dry weight. All the above bar charts were plotted with Mean ± SD. Independent-samples *t*-test analyzed data. The symbols * and ** indicate significant differences at *P* < 0.05 and *P* < 0.01, respectively.

*Pseudophialophora oryzae* and rice were co-cultivated to investigate whether *P. oryzae* promotes rice growth. It was founded that the *P. oryzae* inoculated rice seedlings grew better and stronger than the control plants (Figures 5A,B), exhibiting higher chlorophyll content, shoot height, root length, fresh shoot weight, fresh root weight, and plant dry weight by 24.10, 35.32,19.35, 90.00, 33.3, and 79.17%, respectively (Figures 5C–H). These results indicated that *P. oryzae* possessed a positive capacity for plant growth.

### *Pseudophialophora oryzae* Enhances Resistance Against Rice Blast

**FIGURE 6:**
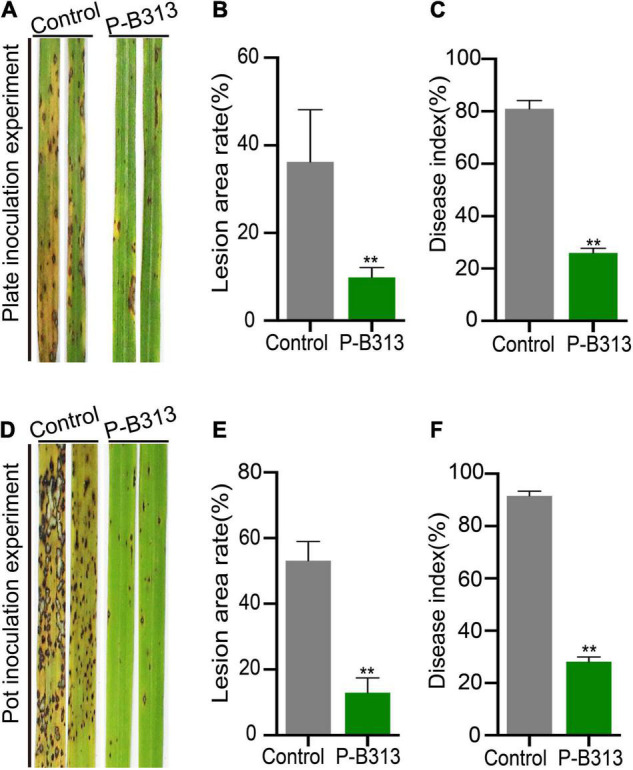
Effect of *P. oryzae* on resistance against rice blast. **(A,D)** The severity of devastating symptoms on the leaves of *P. oryzae*-inoculated rice compared to control in plate and pot experiments, respectively. **(B,E)** An AxioVision image analyzer evaluated the lesion area. Bar charts were plotted with Mean ± SD. **(C,F)** According to the disease classification, the disease index of *P. oryzae*-infected and control-infected rice was calculated. Bar charts were plotted with Mean ± SD from 30 plants, respectively. Independent-samples *t*-test analyzed data. The symbols * and ** indicate significant differences at *P* < 0.05 and *P* < 0.01, respectively.

We then investigated whether *P. oryzae* confers resistance to rice against rice blast under both plate and pot conditions. It was shown that the disease of the control rice plants grown in plates was serious, forming large circular or oval brown spots, disease spots densely covered (Figure 6A). The lesion area rate was 36.23%, and the disease index was 80.95% (Figures 6B,C). In contrast, the disease of rice plants inoculated with *P. oryzae* was relatively mild (Figure 6A), with a 9.91% lesion area rate (Figure 6B). The leaf area of the disease spot was small, accompanied by a few necrotic spots, and the disease index was only 25.92% (Figure 6C). The control effect of *P. oryzae* on rice blast reached 72.65%. Similarly, the disease resistance tests for potted plants were consistent with those for plates (Figure 6D). The lesion area rate of control and treatment was 53.13 and 12.95% (Figure 6E), respectively, and the disease index was 91.54and 28.04% (Figure 6F). The control effect of *P. oryzae* on rice blast reached 75.63% in pots. In conclusion, root colonization of *P. oryzae* can induce systemic disease resistance of hosts and has a positive control effect on rice blast.

### *Pseudophialophora oryzae* Promotes Nutrient Absorption in Rice

**FIGURE 7:**
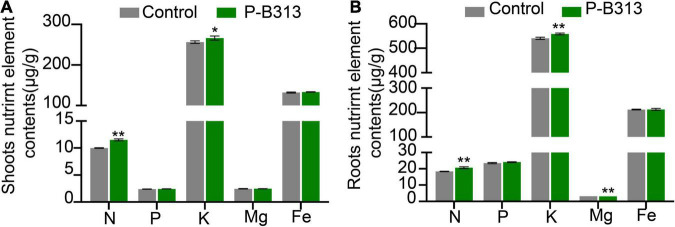
The effect of *P. oryzae* on nutrient content in rice seedling tissues. **(A)** The nutrient content in shoots. **(B)** The nutrient content in roots. Independent-samples *t*-test analyzed data. The symbols * and ** indicate significant differences at *P* < 0.05 and *P* < 0.01, respectively.

Through the analysis of the nutrient element contents in the shoots and roots of rice plants, it was found that after inoculation with *P. oryzae*, the contents of N and K in the shoot tissues of rice plants increased significantly, which increased by 15.28 and 3.88% compared with the control group, respectively (Figure 7A). There was no significant change in P, Mg, and Fe content. Similarly, the contents of elements such as N, K, and Mg in the roots of the treatment group also increased significantly, increasing by 12.35, 3.29, and 0.36%, respectively (Figure 7B). There was no significant change in P and Fe content. Therefore, the root colonization of *P. oryzae* can effectively promote the absorption of nutrient elements in rice roots and increase the content of nutrient elements in the tissues.

### Expression of Genes Related to Nutrient Absorption and Disease Resistance

We analyzed the expression levels of N, P, K, Fe, Mg, and other key genes for nutrient absorption and resistance-related genes. The results showed that the root colonization of *P. oryzae* significantly up-regulated the expression of peptide transporter *OsPTR9* and potassium transporter *OsHAK16*, which were 7.28 ± 0.84 times and 2.57 ± 0.80 times higher than that of the control group, respectively. Genes such as *OsAMT3;2* and *OsMRS2-8* were significantly down-regulated. It can be seen that after *P. oryzae* infects and colonizes rice roots, it can significantly up-regulate the expression of genes related to N and K element absorption, thereby promoting nutrient element absorption (Table 2).

**TABLE 2:** The relative expression of genes related to plant nutrient element absorption.

Gene name	Description	TIGR	Fold change
*OsPTR9*	Peptide transporter	Os06g0706400	7.28 ± 0.84**
*OsAMT3;2*	Ammonium transporter	Os03g0838400	0.22 ± 0.06**
*OsMRS2-8*	Magnesium transporter	Os04g0430900	0.43 ± 0.05**
*OsPT4*	Phosphorus transporter	Os04g0186400	0.68 ± 0.02**
*OsHAK16*	Analogous potassium transporter	Os03g0575200	2.57 ± 0.80*
*OsIRO2*	Iron-related transcription factor 2	Os01g0952800	0.40 ± 0.18**
*OsYSL15*	Iron-phytosiderophore transporter	Os02g0650300	0.27 ± 0.16**

Fold change in relative gene expression were calculated by Mean ± SD. Independent-samples t-test analyzed data. The symbols * and ** indicate significant differences at P < 0.05 and P < 0.01, respectively.

In addition, we found that the root colonization of *P. oryzae* significantly up-regulated the expression of *NAC, OsSAUR2, OsWRKY71, EL5*, and *PR1*α genes, which were 3.04 ± 0.72, 10.37 ± 0.34, 1.98 ± 0.13, 2.10 ± 0.35, and 1.46 ± 0.17 times of the control group, respectively. Compared with the control group, *AOS, POX2*, and *PR1b* were significantly down-regulated by 0.36 ± 0.05, 0.39 ± 0.24, and 0.38 ± 0.16 times. However, the expression levels of *POX1* and *ERF4* were not significantly changed. In conclusion, *P. oryzae* can induce up-regulated expression of some genes representing plant defense response and improve the host systemic disease resistance (Table 3).

**TABLE 3:** The relative expression of selected genes representative for plant defense response.

Gene name	Description	TIGR	Fold change
*NAC*	NAC domain–containing	Os01g0862800	3.04 ± 0.72**
*AOS*	Allene oxide synthase	Os03g0225900	0.36 ± 0.05**
*OsSAUR2*	RNA small auxin-up RNA	Os01g0768333	10.37 ± 0.34**
*OsWRKY71*	Transcription factor	Os02g0181300	1.98 ± 0.13**
*POX1*	Putative peroxidase	Os06g0521500	0.74 ± 0.20
*POX2*	Putative peroxidase	Os06g0521900	0.39 ± 0.24*
*EL5*	N-acetylchitooligosaccharide elicitor-responsive	Os02g0559800	2.10 ± 0.35**
*ERF4*	Ethylene-responsive transcription factor 4	Os04g0610400	2.18 ± 0.98
*PR1*α	Pathogenesis-related gene	Os07g0129200	1.46 ± 0.17**
*PR1b*	Pathogenesis-related gene	Os01g0382000	0.38 ± 0.16**

Fold change in relative gene expression were calculated by Mean ± SD. Data were analyzed by independent-samples t-test. The symbols * and **indicate significant differences at P < 0.05 and P < 0.01, respectively.

## Discussion

**FIGURE 8:**
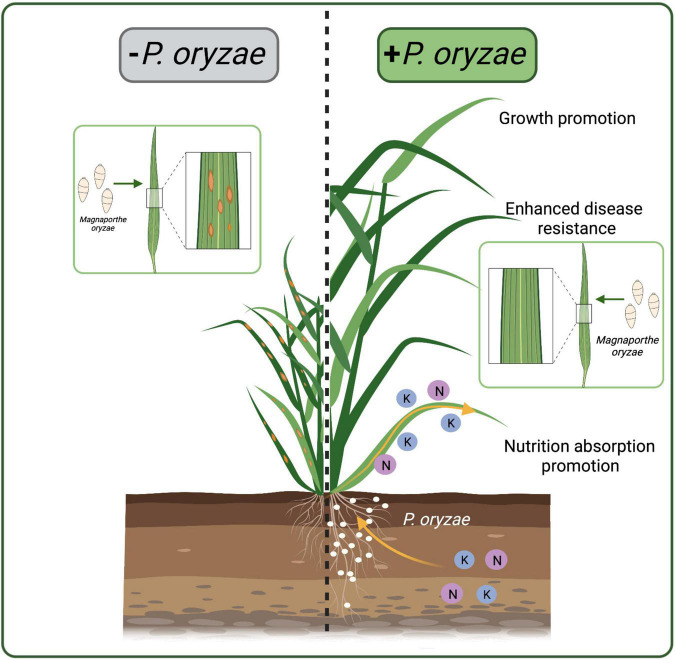
Schematic representations of rice colonized by *P. oryzae*. *P. oryzae* promoted the absorption of N and K elements, promoted rice growth, and enhanced the resistance to rice blast. The white dots represent *P. oryzae*.

Plant roots provide excellent habitats and nutrients for endophytic fungi to help them survive. Endophytic fungi, in turn, protect plants from biotic and abiotic stresses (Verma et al., 2009; Lahrmann et al., 2013; Mitter et al., 2013). Endophytic fungal communities play an important role in adapting wild rice to poor environments. Our study firstly isolated *P. oryzae* from the wild rice roots. There are few reports of *Pseudophialophora* genus, besides *Pseudophialophora* sp. isolated from the grassroots by Luo et al. (2014, 2015). The six-genes phylogeny showed that *P. oryzae* was defined as a singleton in the genus, while *P. panicorum* clustered in another subclade. Morphologically, *P. oryzae* is significantly different from *P. panicorum* (Luo et al., 2014). A new species *P. oryzae* sp. nov was proposed for the first time. And *P. oryzae* was beneficial for rice growth and blast resistance (Figure 8).

The colonization pattern of endophytic fungi is essential for understanding the symbiosis between endophytes and host plants. We found that *P. oryzae* hyphae invaded the root epidermis into the cortex and reached the endodermis but did not approach the stele. This colonization pattern was similar to dark septate endophytes (DSEs) and soil-inhabiting fungi (Maciá-Vicente et al., 2009). Differently, DSEs formed fungal structures, including hyphopodia and microsclerotia (Su et al., 2013), while *P. oryzae* did not form such structures during infection. The fungal proliferation pattern of DSE *H. oryzae* in rice increased firstly and then stabilized (Su et al., 2013). However, the fungal proliferation pattern of *P. oryzae* kept increasing within 20 days, neither causing any disease symptoms.

Endophytes promote plant growth (Rigobelo and Baron, 2021), which is mainly regulated by the levels of plant hormones (Khalmuratova et al., 2021) or promoting plants to obtain essential nutrients (Rigobelo and Baron, 2021). Endophytes can secrete growth-promoting substances such as auxin, cytokinin, gibberellin (Khan et al., 2012), and secondary metabolites (Peters et al., 1998) to regulate hormone levels and promote plant growth and development. Colonization of *Anteaglonium* in blueberry roots changed the metabolism of plant hormones and flavonoids, stimulating blueberries’ growth (Wu et al., 2020). *Alternaria tenuissima* and *Fusarium tricinctum* synthesized auxin and promoted the growth of host plants (Chand et al., 2020). Endophytes also promote nutrient uptake, often including N, P, and K elements critical for plant development (Tan and Zou, 2001). *Xylaria regalis* from cones of *Thuja plicata* could significantly increase the N content of red pepper and thus promote the growth of pepper (Adnan et al., 2018). *Piriformospora indica* improved the accumulation of N and K to improve tomato growth (Ghorbani et al., 2019). In addition, genes related to nutrient absorption also played important roles. *OsPTR9* is a member of the peptide transporter *PTR* gene family. Overexpression of *OsPTR9* could increase the lateral root density of rice, increase the contact area between root and nutrients, fix nitrogen in the atmosphere, promote the absorption of ammonium and the growth of rice (Fang et al., 2013). *OsHAK16* is a member of *HAK/KUP/KT* family and is essential for K absorption (Okada et al., 2008). Overexpression of *OsHAK16* significantly increased K content in rice and improved the stress resistance of rice (Fang et al., 2013). Our results showed that the colonization of *P. oryzae* in the rice roots led to the up-regulation of the expression of *OsPTR9* and *OsHAK16*, which increased the accumulation of N and K in rice and promoted the growth of rice. In addition to enhancing nutrient absorption, whether *P. oryzae* produces hormones or other secretions to promote the growth of the host is still unknown. Therefore, it is necessary to study further the interaction mechanism between *P. oryzae* and rice symbionts.

Endophytes can live in host tissues without causing and adverse symptoms. They can induce plant immune response and improve host disease resistance by regulating genes expression and signal network related to rice defense response (Tsuda and Somssich, 2015). In the defense response of rice, pathogenesis-related (PR) genes are the key genes to induce systemic disease resistance (Asai et al., 2002; Lee et al., 2004; Djamei et al., 2007). *NAC* is one plant-specific transcription factor, which plays an important role in coping with biological and abiotic stresses (Kim et al., 2012; Lv et al., 2016). Several proteins with NAC domain enhanced resistance to *Pseudomonas syringae* infection in tomatoes (Mysore et al., 2002). *OsSAUR2* is an auxin-responsive gene in plants, which has been shown to regulate auxin synthesis and transport, inhibit auxin activity and promote plant immune resistance (Ding et al., 2008; Kant et al., 2009). *EL5* is an N-acetylchitooligosaccharide elicitor response gene in rice, which acts as an *E3* ubiquitin ligase and positively regulates plant immune response (Takai et al., 2002). These reports were consistent with our results that up-regulated expression of *PR1*α, *NAC, OsSAUR2*, and *EL5* can enhance the systemic disease resistance of rice after *P. oryzae* inoculated rice roots. In addition, salicylic acid (SA) (Janda et al., 2020), jasmonic acid (JA) (Barna et al., 2012; Li et al., 2021) and ethylene (ET) (Wang et al., 2019) also play important roles in inducing resistance (Glazebrook, 2005; McDowell et al., 2005; Flors et al., 2008). *AOS* (Gfeller et al., 2010; Xiao et al., 2019) and *ERF4* (Yang et al., 2005) are key genes of JA biosynthesis pathway and ethylene pathway, respectively. Their down-regulated expression indicated that systemic resistance induced by *P. oryzae* was independent of JA and ET signaling pathways. *OsWRKY71* is associated with the SA signaling pathway that regulates the resistance of rice and other gramineous crops to a variety of diseases (Liu X. et al., 2007). The expression of *OsWRKY71* gene was up-regulated by the inoculation of *P. oryzae* in rice. Therefore, the systemic resistance of *P. oryzae* to *M. oryzae* infection may be mediated by SA signaling pathway. Together, our results indicated that *P. oryzae* could induce systemic disease resistance in rice by regulating genes related to rice defense response.

## Conclusion

In conclusion, we isolated an endophytic fungus P-B313 from wild rice and defined it as a new species *P. oryzae* by phylogenetic analysis of six-genes. After co-culture with rice, the colonization pattern of *P. oryzae* was that hyphae invaded from the epidermis to the inner cortex but not into the stele. *P. oryzae* can also promote nitrogen and potassium elements absorption in rice, significantly promote rice growth, and enhance the systemic resistance against rice blast. It can be further developed as a new biogenic agent for agricultural production, providing a new approach for the biocontrol of rice blast.

## Data Availability Statement

The datasets presented in this study can be found in online repositories. The names of the repository/repositories and accession number(s) can be found in the article/Supplementary Material.

## Author Contributions

J-NZ and Z-ZS contributed to experimental design. J-NZ, Y-JY, M-DD, and Y-LZ contributed to experiments. J-NZ, X-JL, and LW contributed to data analysis and scripts. F-CL, X-HL, and Z-ZS supplied experimental conditions. J-NZ, Y-JY, Z-ZS, and F-CL wrote the manuscript. All authors contributed to the article and approved the submitted version.

## Conflict of Interest

The authors declare that the research was conducted in the absence of any commercial or financial relationships that could be construed as a potential conflict of interest.

## Publisher’s Note

All claims expressed in this article are solely those of the authors and do not necessarily represent those of their affiliated organizations, or those of the publisher, the editors and the reviewers. Any product that may be evaluated in this article, or claim that may be made by its manufacturer, is not guaranteed or endorsed by the publisher.
