# Supplementary material for: A New Species in Pseudophialophora From Wild Rice and Beneficial Potential
Source: Front Microbiol. 2022 Mar 11;13:845104. doi: 10.3389/fmicb.2022.845104 (PMC8963453; doi:10.3389/fmicb.2022.845104)
Supplement: Supplementary file 1 [file Data_Sheet_1.docx]

| **Table S1** Primers used in this study | |
| --- | --- |
| **Primer name** | **Primer sequences (5’-3’)** |
| ITS-F | TCCGTAGGTGAACCTGCGG |
| ITS-R | TCCTCCGCTTATTGATATGC |
| LSU-F | GTACCCGCTGAACTTAAGC |
| LSU-R | TCCTGAGGGAAACTTCG |
| RPB1-F | GARTGYCCDGGDCAYTTYGG |
| RPB1-R | CCNGCDATNTCRTTRTCCATRTA |
| SSU-F | GTAGTCATATGCTTGTCTC |
| SSU-R | CTTCCGTCAATTCCTTTAAG |
| MCM7-F | CAGGACTGCAAGGACAAC |
| MCM7-R | GGATCTTCATGCCGTCAC |
| TEF1-F | GCYCCYGGHCAYCGTGAYTT |
| TEF1-R | ATGACACCRACRGCRACRGTYTGYAT |
| *Actin-F* | GAGCTACGAGCTTCCTGATGGA |
| Actin-R | CCTCAGGGCAGCGGAAA |
| *OsActin-F* | GAGTATGATGAGTCGGGTCCAG |
| *OsActin-R* | ACACCAACAATCCCAAACAGAG |
| OsPTR9-F | TCTCGGGCGTACAGGTTT |
| OsPTR9-R | GGAGGTCGGATGGACTTATT |
| *OsAMT3;2-F* | TGTACTTCCAGTGCGTGTTC |
| *OsAMT3;2-R* | ACGGTGTAGGAGAAGGTGAG |
| *OsMRS2-8-F* | GGCAAAGTTCAAAAGGTCAGG |
| *OsMRS2-8-R* | TCTCCGTTAGGTGCAATGC |
| OsPT4-F | CTAGTGTACCAAACAAAATTACA |
| OsPT4-R | TGGCATTTATAATATCAACAGTAAC |
| *OsHAK16-F* | TGTGCTAAACCCTTCAGTCTC |
| *OsHAK16-R* | CAATCTTGTCAGTGCCAAACC |
| *OsIRO2-F* | GGCATGGCTCCCATCGT |
| *OsIRO2-R* | AACAAGCTGACCTGAACCATGA |
| *OsYSL15-F* | CAATGTGCCAGGGAGCTATAA |
| *OsYSL15-R* | TACGACCAAAGCCTTTCTTAGG |
| *NAC-F* | CGTCATCCCCGTCTTCG |
| *NAC-R* | GGCTGAACTCGCTCCTCA |
| *AOS-F* | ACCGTCGTCCGCATCAAC |
| *AOS-R* | TGCCATAGGAGCCACAGG |
| *OsSAUR2-F* | TCTCATTACCGAAGCACACC |
| *OsSAUR2-R* | CGGCCTTCATCTCAGCTAC |
| *OsWRKY71-F* | AGATGGCGATGACGCTGAC |
| *OsWRKY71-R* | AGCAATCGTCAATCCTTGGT |
| *POX1-F* | TCGCTTCGGTTGCTCTGC |
| *POX1-R* | TTGATGGCGTCGATCACG |
| *POX2-F* | TTCGGTCACGAGGAGTTCA |
| *POX2-R* | AAGGATGGAGGCATCACAA |
| *OsEL5-F* | TCGGCTCCCACTCCACCTG |
| *OsEL5-R* | TCGGGGATCTCGATCACCAG |
| *ERF4-F* | CAGGTACGCGGCGGAGATC |
| *ERF4-R* | GGGGTCGAGGAACAGGAACG |
| *OsPR1a-F* | GTATGCTATGCTACGTGTTTATGC |
| *OsPR1a-R* | GCAAATACGGCTGACAGTACAG |
| *OsPR1b-F* | ACGCCTTCACGGTCCATAC |
| *OsPR1b-R* | AAACAGAAAGAAACAGAGGGAGTAC |

| **TABLE S2** Disease classification standard | |
| --- | --- |
| **Disease value** | **Disease spot area** |
| 0 | No disease |
| 1 | Disease spot number <5, Disease spot length<1cm |
| 3 | Disease spot number 6-10, Disease spot length>1cm |
| 5 | Disease spot number 11-25, Lesion area 10%-25% |
| 7 | Disease spot number >26, Lesion area 25%-50% |
| 9 | Disease spots into a piece，Lesion area >50% or All dead |
